# Anti‐*Acanthamoeba* Activities of Silica Nanoformulated Quercetin Compound Derived From *Annona muricata* L. Leaves

**DOI:** 10.1155/sci5/5902580

**Published:** 2026-09-01

**Authors:** Hazel Anne Tabo, Siriphorn Chimplee, Diana Mendonça, Ryan V. Labana, Tajudeen O. Jimoh, Tooba Mahboob, Norbel Tabo, Arizaldo Castro, Darylle Cesar G. Hilapo, Feibing Huang, Qiongdan Zhang, Ling Liang, Rachasak Boonhok, Bin Li, Wei Wang, Maria de Lourdes Pereira, Cristina Salibay, Shanmuga Sundar Saravanabhavan, Muhammad Nawaz, Veeranoot Nissapatorn

**Affiliations:** ^1^ Biological Sciences Department, College of Science, De La Salle University-Dasmariñas, Dasmariñas City, Cavite, Calabarzon, Philippines; ^2^ School of Languages and General Education, Walailak University, Nakhon Si Thammarat, Thailand, wu.ac.th; ^3^ CICECO, Universidade de Aveiro, Aveiro, Portugal, ua.pt; ^4^ Institute of Biosciences, Humanities and Exact Sciences (Ibilce), São Paulo State University (UNESP), São José Do Rio Preto 15.054-999, State of São Paulo, Brazil, unesp.br; ^5^ Center for Health Science, Research Institute for Science and Technology, and Department of Biology, College of Science, Polytechnic University of the Philippines, Sta. Mesa, Manila, Philippines, pup.edu.ph; ^6^ The Graduate School, University of Santo Tomas, España Blvd Sampaloc, Manila, Philippines, ust.edu.ph; ^7^ Department of Microbiology, Icahn School of Medicine at Mount Sinai, New York, New York, USA, mountsinai.org; ^8^ Faculty of Pharmaceutical Sciences, UCSI University, Cheras, Federal Territory of Kuala Lumpur, Malaysia, ucsiuniversity.edu.my; ^9^ School of Medical Technology, Emilio Aguinaldo College-Cavite Campus, Dasmariñas City, Cavite, Calabarzon, Philippines; ^10^ Faculty of College of Health Sciences Education, Olivarez College, Parañaque, Metro Manila, Philippines; ^11^ School of Pharmacy, Hunan University of Chinese Medicine, Changsha, Hunan, China, hnctcm.edu.cn; ^12^ School of Allied Health Sciences, Walailak University, Nakhon Si Thammarat, Thailand, wu.ac.th; ^13^ Department of Medical Sciences, Universidade de Aveiro, Aveiro, Portugal, ua.pt; ^14^ Department of Biotechnology, Aarupadai Veedu Institute of Technology, VMRF (DU), Paiyanoor, Chennai, Tamil Nadu, India, avit.ac.in; ^15^ Department of Nano-Medicine Research, Institute for Research and Medical Consultations (IRMC), Imam Abdulrahman Bin Faisal University, Dammam, Saudi Arabia, iau.edu.sa; ^16^ Futuristic Science Research Center–School of Science and World Union for Herbal Drug Discovery (WUHeDD), Walailak University, Nakhon Si Thammarat, Thailand, wu.ac.th

**Keywords:** *Annona muricata*, anti-*Acanthamoeba*, nanocompound, nanoformulation, phytocompound, quercetin

## Abstract

*Acanthamoeba* spp., free‐living amoebae (FLA), are the causative agents of *Acanthamoeba* keratitis (AK) and granulomatous amoebic encephalitis (GAE). These diseases have limited treatment options; therefore, drug discovery remains a critical priority. This study evaluated the leaf extract of *Annona muricata* L., which contains quercetin, formulated in silica nanoparticles against *Acanthamoeba* species. The anti‐*Acanthamoeba* effect of the nanoformulation was determined by its MIC and percentage viability. Scanning electron microscopy confirmed the nanocompound‐directed effect on trophozoites and cysts. The nanoquercetin exhibited anti‐*Acanthamoeba* activity, showing greater effectiveness against the trophozoites of *Acanthamoeba polyphaga* ATCC 30461 (AP) (MIC > 2 mg/mL) compared to cysts (MIC > 8 mg/mL) and against *A. triangularis* WU19001 (AT) trophozoites (MIC > 4 mg/mL) compared to cysts (MIC > 8 mg/mL). It also showed reduced activity against *Acanthamoeba castellanii* ATCC 30010 (AC3) trophozoites (MIC > 4 mg/mL) compared to cysts (MIC > 8 mg/mL) and *A. castellanii* ATCC50739 (AC5) trophozoites (MIC > 4 mg/mL) compared to cysts (MIC > 8 mg/mL). The anti‐*Acanthamoeba* activity of *A. muricata* on all four species was compared to chlorhexidine, which had MIC values of 0.008 mg/mL for AC3 and AT trophozoites and 0.016 mg/mL for AC5 and AP trophozoites. For cysts, chlorhexidine exhibited MIC values of > 0.0128 mg/mL for AC3 and AP, 0.032 mg/mL for AC5, and 0.064 mg/mL for AT. All MIC values demonstrated inhibition of *Acanthamoeba* species with reduced viability exceeding 90%. SEM findings revealed morphological changes in *Acanthamoeba*, indicating parasitic inhibition. Quercetin‐loaded silica nanoparticles demonstrated anti‐*Acanthamoeba* activity with low cytotoxicity. This indicates the potential of quercetin as a nano‐enhanced natural therapeutic or contact lens‐care agent. This study is limited by its in vitro design, small parasite strain panel, and potential nanoparticle variability. This highlights the need for in vivo validation, ocular/contact lens model testing, and formulation optimization for improved cysticidal efficacy.

## 1. Introduction

*Acanthamoeba* spp. are opportunistic, free‐living pathogens widely distributed in the environment [1]. They have been detected on the nasal epithelial surfaces of healthy humans, particularly during windy periods [2]. Currently, 25 named species of *Acanthamoeba* are recorded in the National Center for Biotechnology Information (NCBI) taxonomy database [3], along with 20 published genotypes, with T4 being the major genotype associated with human infections [4]. Certain genotypes are responsible for human diseases, such as the debilitating *Acanthamoeba* keratitis (AK), which can cause blindness, and the fatal granulomatous amoebic encephalitis (GAE) [5]. AK is characterized by severe ophthalmalgia, photophobia, blue‐red vision, and blood extravasation [6]. The clinical symptoms of GAE include headaches, fever, and neurological disorders, such as vision impairment, personality changes, and coma [7].

Chlorhexidine (CHX) and polyhexamethylene are commonly used in the treatment of AK. However, their daily and long‐term use can lead to adverse effects, including impaired corneal regeneration, cataract formation, iris atrophy, corneal ulceration, glaucoma, hyperemia, and photophobia [8]. Corticosteroids may exacerbate keratitis when the cornea is infected with *Acanthamoeba castellanii*, as shown in vivo in Japanese white male rabbits [9]. Current therapeutic options are associated with toxic side effects to human keratocytes and exhibit either negligible or low cysticidal efficacy [10]. Consequently, there is an urgent need to identify new drugs and drug delivery systems capable of crossing biological barriers while exhibiting minimal toxicity to human cells.

As the demand for medicines continues to rise due to population growth and increasing prevalence of diseases, researchers are actively seeking new and more effective drug sources. The use of medicinal plants for treating various ailments has long attracted significant global attention [11]. In Southeast Asia, at least 110 plant species, particularly from the Lamiaceae and Asteraceae families, have been reported to exhibit anti‐*Acanthamoeba* activity [12]. In the Philippines, recognized plants with medicinal value such as *Annona muricata*, *Carica papaya*, and *Moringa oleifera* have emerged as particularly promising due to their rich phytochemical content. *A*. *muricata* has demonstrated efficacy against trophozoites and cysts, whereas *C. papaya* and *M. oleifera* are noted for their antimicrobial activities [13]. The medicinal applications of these plants have garnered attention for their bioactive properties [14]. Their predominant compounds, such as acetogenins, exhibit neurotoxicity in in vitro and in vivo studies [14]. Additionally, other compounds demonstrate various activities, including anti‐inflammatory, anticancer [15], antimicrobial, antiprotozoal, antioxidant, insecticidal, larvicidal, selective cytotoxicity toward tumoral cells, anxiolytic, antistress, antiulcer, wound healing, anti‐icteric, hepatoprotective, and hypoglycemic effects [14].

Quercetin, a flavonoid commonly found in many medicinal plants, including *A*. *muricata*, has been studied for its antimicrobial and antiparasitic potential. However, its anti‐*Acanthamoeba* effects have yielded mixed results in previous studies. For instance, Anwar et al. [16] reported that quercetin alone showed limited trophozoiticidal activity against *A. castellanii,* but its efficacy improved markedly when conjugated to silver nanoparticles (AgNPs) [16]**.** Nanoparticles (NPs), a class of nanomaterials, are notable for their applications in the treatment and control of various diseases [17]. Metal NPs, such as silver (Ag) [18] and gold (Au), have shown potential in treating infections caused by *Cryptosporidium*, *Leishmania*, and *Toxoplasma* [19]. Other NPs, such as copper (Cu), silicon (Si) [20], titanium (Ti), and zinc (Zn), have demonstrated antimicrobial activity against a broad range of pathogens [21]. NPs are characterized by ease of synthesis, improved drug solubility and stability, and the ability to modulate drug release in response to controlled stimuli, such as light, pH, and heat [22]. However, some NPs, particularly those with high toxicity potential, like silver or carbon nanotubes, raise environmental and health concerns, making them less favorable. In contrast, NPs designed for targeted drug delivery using biocompatible materials are considered more advantageous [22]. In a separate study, Abdel‐Hakeem et al. [23] demonstrated the therapeutic effects of quercetin–AgNPs in a murine model of *Acanthamoeba polyphaga* encephalitis [23], highlighting the potential of nanoformulated flavonoids as antiamoebic agents. In contrast, Lê et al. [24] screened a series of flavonoids and reported no detectable anti‐*Acanthamoeba* activity for quercetin [24]. These findings indicate that while quercetin possesses promising pharmacological properties, its direct amoebicidal effect remains inconsistent and may depend on the delivery system, compound stability, or NP carrier used. Hence, there is a continuing need to explore alternative nanocarriers, such as biocompatible silica NPs (SNPs), to enhance quercetin’s efficacy while minimizing toxicity. SNPs have gained significant recognition from the scientific community due to their diverse physicochemical properties [25]. Typically, inexpensive to produce on a large scale, SNPs are hydrophobic and exhibit excellent surface area, pore volume, and biocompatibility, making them suitable for a wide variety of applications [26, 27]. In parasitology, SNPs have been successfully applied for the treatment of *Plasmodium* infections [28]. Quercetin’s therapeutic potential, however, is limited by poor solubility and instability under physiological conditions. In the current study, SNPs were therefore chosen as the delivery system because they improve quercetin’s solubility, stability, and controlled release, while remaining nontoxic and safer compared with metal‐based nanocarriers. These properties make silica nanoformulation a promising vehicle to enhance quercetin’s anti‐*Acanthamoeba* efficacy.

This study aimed to (1) characterize silicon dioxide (SiO_2_ NPs); (2) determine the anti‐*Acanthamoeba* activity of SiO_2_ NPs; and (3) evaluate the antiadhesive potential of crude and purified SiO_2_ NPs, derived from the bioactive compound of *A*. *muricata* Linn., against *Acanthamoeba* spp. in vitro*.*

## 2. Materials and Methods

### 2.1. Plant Material and Extract Preparation

**FIGURE 1:**
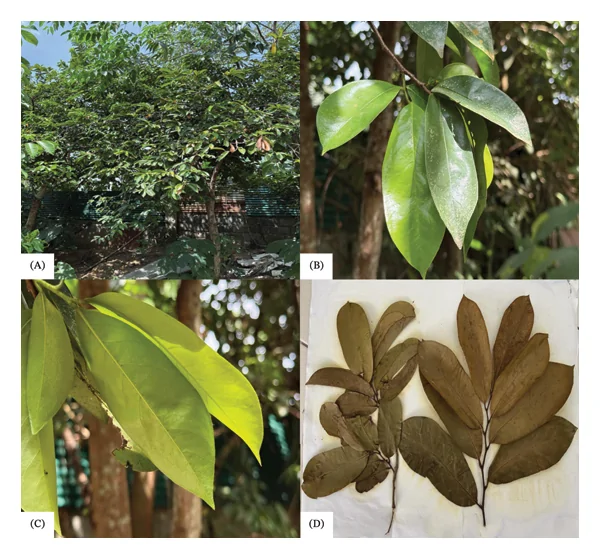
*A. muricata* (guyabano) tree and leaves. (A) Tree selected for collection of leaves; (B) anterior part of fresh mature leaves; (C) posterior part of fresh mature leaves; (D) herbarium voucher specimen.

Fresh, mature, and healthy leaves of *A*. *muricata* (Figure 1) were collected from San Pablo City, Laguna, Philippines. The leaves were thoroughly cleaned with distilled water to remove debris, then placed in an ice chest and transported to the laboratory for processing. Species identification was confirmed by a botanist in the Philippines. The air‐dried leaves were pulverized using a food processor. Two hundred grams (200 g) of the powdered leaves was soaked in 800 mL of 95% ethanol for 5 days at room temperature (25°C ± 2°C) with occasional stirring. The extracts were then filtered through filter paper (GE Healthcare Life Sciences, IL, USA) and processed using a rotary evaporator (Buchi, New Castle, USA).

The plant voucher specimen has been verified and authenticated based on botanical accessions and literature by Dr. Sandra Yap, a botanist curator from Far Eastern University Herbarium (FEUH), Manila, Philippines, with the specimen accession number 2025‐104A as *A*. *muricata* L. from the family Annonaceae.

### 2.2. GC–MS, HPLC, and NMR Profiling of Ethanolic Leaf Extract of *A. muricata*

Based on a preliminary crude extract assessment against *Acanthamoeba* spp., *A. muricata* was selected to be subjected to GC–MS characterization. Briefly, prepared samples of 100 μL were mixed with 1900 μL of ethanol, transferred to a GC vial, and injected into the GC–MS. The sample composition was determined and semiquantified using a GC–MS machine (Shimadzu QP2010 Ultra, Japan). The sample was administered into the GC–MS injector port at 270°C in split mode at a 5.0 split ratio. Analytes were carried through a DB5‐MS column (30 m, 0.25 mm ID, 0.2‐ μm film thickness) with helium as a gas carrier and run at 45 cm/s linear velocity. The oven temperature and time settings were adjusted initially to 50°C for 5 min, then increased to 270°C at a rate of 15°C/min, and held for 15 min. The eluted analytes were subjected to electron ionization at a source temperature of 230°C with an interface temperature of 270°C. The mass spectrometry dataset was in scan mode with a 3‐min solvent cut time, and the m/z range was set at 40–800 with an event time of 0.30 s. To identify compounds, the chromatogram peaks using the highest similarity index, with a similarity index greater than 80%, from the NIST 08 library search and similar peak retention time (RT) from the PIPAC database were used as reference identification.

Silica gel column chromatography and HPLC were used to purify and isolate the target compound at the IMMRI Laboratory, Hunan University of Chinese Medicine. An MCM‐41 silica slurry (methanol:water, 1:1) was prepared, packed into a column, and topped with sand. The ethanolic crude extract of *A. muricata* was dry‐loaded using MCI gel and applied to the column. Elution with methanol:water was performed, with fractions monitored by TLC and combined before solvent removal via rotary evaporation (52°C, 75 rpm). The dried sample was redissolved in ethanol and injected (3 μL) into HPLC (210–250 nm). Compound identification was confirmed using ^1^H and ^13^C NMR. Results were verified by a registered chemist (Assoc. Prof. Gideon Legaspi, DLSU‐D), confirming the presence of quercetin.

### 2.3. Preparation of SiO_2_ NPs

Tetraethyl orthosilicate (TEOS), ethanol, and ammonia were used as the silica precursor, common solvent, and catalyst, respectively. Briefly, 28 mL of ethanol and 6 mL of ammonia were added to 22 mL of deionized water (dH_2_O) and stirred using a magnetic stirrer at room temperature (RT; 26°C) for 5 min to achieve homogeneity. Three milliliters of TEOS was then added to the solution, which was tightly covered with foil. The mixture was stirred again for 20 h. The solution was centrifuged at 6000 rpm for 5 min, and the pellets were washed with 40 mL of dH_2_O. The solution was centrifuged again at 6000 rpm for 5 min, and the pellets were dissolved with 30 mL ethanol. The ethanol solution was centrifuged again at 6000 rpm for 5 min, and the pellets were oven‐dried at 60°C for 24 h. The resulting white powder, representing the bare or nano‐silica blank (NSB), was then oven‐dried at 200°C for 4 h and calcined for 5.5 h at 550°C. The final product was transferred to a desiccator until further use [29].

### 2.4. Quercetin Loading and Release Profiling of SiO_2_ NPs

A total of 100–150 mg of SiO_2_ NPs as blank or bare NPs was soaked in a saturated solution of a natural product for 24–48 h with stirring to ensure adequate loading. The mixture was then centrifuged, and the supernatant was analyzed using a UV–visible spectrophotometer (SpectraMax, M3, Molecular Devices, Washington, DC, USA) [29]. To study the release of the natural product, 10–15 mg of the natural product–loaded SiO_2_ NPs was dispersed in phosphate‐buffered saline (PBS) at pH 7.4 and maintained at 37°C while stirring. At various time intervals, 3 mL of the solution was removed, and 3 mL of fresh PBS was added to maintain the overall volume. The removed solution was then centrifuged and analyzed using a UV–visible spectrophotometer [29].

### 2.5. Characterization of Natural Product–Loaded SiO_2_ NPs

The morphology and size of natural product–loaded SiO_2_ NPs were determined using transmission electron microscopy (TEM) (FE‐TEM, JEOL, Tokyo, Japan). The phases of NPs were analyzed with energy‐dispersive X‐ray spectroscopy (EDX) using Cu‐Kα radiation (*λ* = 1.5418 Ǻ) at a scanning speed of 1° per minute over a range of 10°–80°. To identify the functional groups in both the natural product–loaded SiO_2_ NPs and the unloaded material, Fourier transform infrared (FTIR) analysis using a PerkinElmer (Boston, Massachusetts, USA) spectrum capture analysis was performed. Additionally, the zeta potential (Z‐potential) and particle size distribution of natural product–loaded SiO_2_ NPs were evaluated using FTIR [29–31]. To determine the loading efficiency, we determined the following: the nanoformulation yield, encapsulation efficiency (EE), and drug loading content. The natural product–loaded SiO_2_ NPs were initially centrifuged with settings at 1800 rpm and 4°C for 30 min to separate the free compound from the encapsulated form. Both the natural product–loaded SiO_2_ NPs and the unloaded free drug compound solutions were collected, air‐dried, and further freeze‐dried for approximately 24 h to promote their powder forms. Both samples were separately weighed to determine the mass yield. The EE and drug loading content were determined by dissolving the nanoformulated SiO_2_ powder in 95% ethanol. The drug concentration was measured using spectrometry at 570 nm absorbance. The EE and drug load content were also calculated using Equation (1) below, respectively [61, 69]:

(1) % drug entrapment=mass of total drug−mass of free drugmass of total drug×100.

Then, 15 mg of the quercetin‐loaded SiO_2_ NPs was suspended in 40 mL of PBS, calibrated to pH 7.4, and stirred continuously for 72 h; thereafter, the absorbance was read at 570 nm wavelength at periodic intervals to determine its drug release after 24 h using the initial burst method [29, 61, 69]. Aside from these characterization methods, the Z‐potential character of the natural product quercetin‐loaded SiO_2_ particles (nanoformulated quercetin [NQT]) is related to the drug release potential of the NQT NPs and the molecular stability of the nanoformulation that includes the drug that is loaded, retained, and released at a given time. To quantify % relative drug release over time (from 0 h [initial] to 72 h [final]), the absorbance of NQT NPs was read at 570 nm at multiple time points, i.e., 24, 48, and 72 h.

### 2.6. *Acanthamoeba* Cultivation

*Acanthamoeba* culture was conducted in compliance with the biosafety guidelines for scientific research at Walailak University, Nakhon Si Thammarat, Thailand (Ref. No. WU‐IBC‐66‐020). Cultivation of *Acanthamoeba* was performed based on methods described previously by Mitsuwan et al. and Sangkanu et al. [13, 32]. *A*. *castellanii* ATCC50739 (AC5) and *A. triangularis* WU19001 were provided by Assistant Professor Dr. Rachasak Boonhok from the Department of Medical Technology, School of Allied Health Sciences, Walailak University, Thailand, and *A. polyphaga* ATCC30461 (AP) and *A. castellanii* ATCC30010 (AC3), purchased as pathogenic forms from the American Type Culture Collection (ATCC), were used in this study. The *Acanthamoeba* spp. trophozoites were grown in Peptone Yeast Extract Glucose Broth (PYG) medium (HiMedia Laboratories Pvt. Ltd., Mumbai, India) containing proteose peptone 0.75% (w/v), yeast extract 0.75% (w/v), and glucose 1.5% (w/v) at RT (26°C) and centrifuged at 4000 rpm for 5 min in fresh PYG medium prior to further testing. Cysts were cultivated using Neff’s medium containing 7.4 g KCl, 2.4 g ammediol, 0.08 g NaHCO_3_, 1.98 g MgSO_4_•7 H_2_O, and 0.05 g CaCl_2_•2 H_2_O and collected after an incubation period of 7 days.

### 2.7. Anti‐*Acanthamoeba* Assays

#### 2.7.1. Preliminary In Vitro Screening of Plant Against *Acanthamoeba* spp.

The crude extract (200 mg/mL) of *A. muricata* was dissolved in 99.9% dimethyl sulphoxide (DMSO; Sigma Chemical Co, St Louis, MO, USA). Negative controls were treated with 2%–4% DMSO, and positive controls (CHX; 0.008 and 0.016 mg/mL) were added to each set of experiments. The assays were performed in a 96‐well microplate. The final concentrations of trophozoites and cysts were adjusted to 2 × 10^5^ cells/mL. Fifty microliters (50 μL) of the calibrated trophozoite/cyst suspension and the test compound (4 and 8 mg/mL) were used in the minimum inhibitory concentration (MIC) assay. After 24 h, 50 μL of parasite–compound suspension was mixed with 50 μL of 0.2% trypan blue. The unstained (colorless; viable) and stained (blue; nonviable) cells were counted separately using a hemocytometer (Boeco, Hamburg, Germany) under an inverted microscope (Nikon ECLIPSE TE2000‐S; Nikon, Tokyo, Japan). The relative percentage of cell viability was calculated as follows: (mean of the treated parasite/mean of the negative control) x 100 [13].

#### 2.7.2. MIC Assay

Regarding the best potentials of *A. muricata* on tested *Acanthamoeba* spp., the relevant major compound, quercetin, derived from the extract was further studied for SNPs nanoformulation. The MIC assay was used to assess the anti‐*Acanthamoeba* activities of *A. muricata* crude extract, pure quercetin (PQT), NQT, and NSB toward both *Acanthamoeba* trophozoite and cyst forms. A stock solution of the test compounds, at a concentration of 200 mg/mL, was made initially with 99.9% DMSO. NP samples of NSB and NQT were frozen at 4°C and later used as per requirement. The assay was performed in 96‐well flat‐bottom polystyrene microplates (0.33 cm^2^ well surface area, 0.075–0.2 mL volume; VWR International, USA) according to the protocol outlined by Mitsuwan et al. [13]. Trophozoites of *A. castellanii* (ATCC30010 and ATCC50739), *A. polyphaga* (ATCC30461), and *Acanthamoeba triangularis* (WU19001) were grown in PYG medium, and cysts were induced and cultivated in Neff’s encystment medium. For each assay, 50 μL of suspension of parasites (2 × 10^5^ cells/mL) was placed in the wells with 100 μL of serially diluted test compounds. The concentration ranges were 0.0156–8 mg/mL for *A. muricata* extract (AME) and quercetin formulations, and 0.001–0.128 mg/mL for CHX, which was used as the positive control. DMSO was used as the negative control at 2% for trophozoites and 4% for cysts. After 24‐h incubation at RT (26°C), 50 μL per well was mixed with 50 μL of 0.2% trypan blue and counted under an inverted microscope in a hemocytometer. Viable (unstained) and dead (blue‐stained) cells were enumerated. MIC was established as the lowest concentration of the test compound to show ≥ 90% inhibition of parasite viability compared to the control DMSO. MIC values were also divided into minimum trophocidal inhibitory concentration (MTIC) and minimum cysticidal inhibitory concentration (MCIC) for simplicity in reporting. Compounds that showed ≥ 90% inhibition in either of these forms were used for subsequent parasiticidal and mechanistic studies. Viability was calculated using the following formula:

(2) % viability=number of viable treated Acanthamoeba cellnumber of viable untreated Acanthamoeba cell×100.

#### 2.7.3. Minimum Parasiticidal Concentration (MPC) Assay

Following Equation (1), the MPC preparation was adopted from methods described by Sangkanu et al. (2021) and Chimplee et al. [32, 33]. Two‐fold serial dilution was performed with 100 μL as final concentrations of 16, 8, 4, 2, 1, and 0.5 mg/mL of NSB, NQT, PQT, and crude AME were set, and then 2 × 10^5^ cells/mL of trophozoites and cysts were administered into each well of a 96‐well microplate. CHX was set at 0.008 and 0.016 mg/mL, and DMSO (2% for trophozoites and 4% for cysts) was used as the positive and negative controls, respectively. After 24‐h incubation for MIC, viability observation was extended to 72 h for trophocidal and cysticidal activities of NSB, NQT, and PQT. After 72 h, respective cell suspensions mixed with trypan blue dye were placed on a hemocytometer. Counting of viable and nonviable cells to yield parasiticidal activities of NSB, NQT, and PQT against respective *Acanthamoeba* trophozoites and cysts was determined in the same manner as for the prior MIC using the percentage of trophozoite or cyst viability, defined as (mean of the treated trophozoite or cyst/mean of the control) × 100. The MPC were determined separately for trophozoites, which is the MTIC), and for cysts, which is the MCIC, defined as the lowest concentration with > 99.99% inhibition of viable growth after 72 h or 3‐day observation [32, 33].

### 2.8. Scanning Electron Microscopy (SEM)

The effects of NSB, NQT, and PQT NPs on *A. triangularis* and multiple strains of *A. polyphaga* trophozoites and cysts were determined through a SEM study. *Acanthamoeba* trophozoites and cysts were treated with MIC concentrations of NQT (0.25 mg/mL and 4 mg/mL). After 24 h of treatment, cells were collected by centrifugation at 4000 rpm for 5 min and then resuspended in PBS (Oxoid Holdings, Hampshire, UK). Negative controls for the trophozoites and cysts were 2% and 4% DMSO. Samples were fixed with 2.5% glutaraldehyde for 12 h. Samples were further dehydrated using a series of graded alcohols (20%, 40%, 60%, 80%, 90%, and 100% ethanol), mounted on aluminum stubs, and allowed to dry in a critical point dryer (EMS/K850, Quorum, Laughton, UK). Samples were then coated with gold particles, and the morphology of *Acanthamoeba* trophozoites and cysts after treatment was examined under SEM (SEM‐Zeiss, Munich, Germany) at the Scientific and Technological Equipment Center, Walailak University, Nakhon Si Thammarat, Thailand.

### 2.9. Antiadhesion Assay

The activity of the AMEs and NSB, NQT, and PQT NPs on the adhesion of the *Acanthamoeba* spp. was carried out using a 96‐well polystyrene microplate. Cultures of *Acanthamoeba castellanii* ATCC30010, *A. castellanii* ATCC50739, *A. polyphaga* ATCC30461, and *A. triangularis* WU19001 were grown in the medium supplemented with the extracts at 1/2MIC, 1/4MIC, 1/8MIC, and 1/16MIC. The MIC of CHX and multipurpose solution (MPS; Duna, Alcon Laboratories, TX, USA) were used as positive controls, while 1% DMSO was used as a negative control. Samples were incubated at 28°C for 24 h. Nonadherent cells were eliminated by removing the old medium. Later, the microplates were washed twice with PBS and air dried. Then, samples were stained with a 0.1% crystal violet assay for 30 min. Subsequently, the plates were washed twice with sterile distilled water. The samples were air dried for 30 min at RT (26°C). A total of 100 μL of 99.9% DMSO was added to dissolve the stained cells. The plates were measured at an optical density of 570 nm using a microplate spectrophotometer. The activity of the extracts on the adhesion of *A. triangularis* and *A. polyphaga* was reported as a relative percentage of the adhesion [13] using Equation (3).

(3) % Inhibition=control ODnegative control−Test ODcontrol ODnegative control×100.

### 2.10. Minimum Cytotoxic Concentration (MCC) Using Vero Cells

The cytotoxicity assay was performed using the 3‐[4,5‐dimethylthiazol‐2‐yl]‐2,5 diphenyl tetrazolium bromide (MTT) method by Chimplee et al. [33]. The crude leaf extract of *A. muricata* and nanoformulations were tested for cytotoxicity using Vero cells (ECACC 84113001, RRID: CVCL 0059, Salisbury, UK) upon kind provision of Associate Professor Dr. Chuchard Punsawad (School of Medicine, Walailak University). Cells were cultivated in Dulbecco’s Modified Eagle’s (DMEM) medium (Merck KGaA, Darmstadt, Germany) with 10% fetal bovine serum (Sigma Aldrich, St. Louis, USA) and supplied with 1% penicillin–streptomycin (Thermo Fisher Scientific, MA, USA). Incubated Vero cells were set at 37°C in an optimal environment with 5% CO_2_ to reach 90% confluence, and then 2.5% trypsin–ethylenediaminetetraacetic acid (EDTA) (Thermo Fisher Scientific, MA, US) was added to promote cell detachment and reincubated for 5–10 min. In a 96‐well microplate, calibrated cells for seeding were set at 1.5 × 10^4^ cells/mL and allowed to settle for 24 h. An initial 2 mg/mL concentration of the plant extract and nanoformulation was added to the first well, and serial two‐fold dilutions were performed to achieve desired concentrations of 1, 0.5, 0.25, 0.125, 0.0625, 0.031 and 0.015 mg/mL. Then, 1% DMSO was used as the negative control and incubated for 24 h. Briefly, 100 μL of 0.5 mg/mL MTT (Thermo Fisher Scientific, MA, US) was added to the plates and followed by an hour of reincubation. The reagent was then discarded, and a small amount of 99.9% DMSO was added before the absorbance reading. In a microplate spectrophotometer reader, absorbance readings were obtained at 570 and 650 nm to determine the percent survival as presented in Equation (4).

(4) % Cell survial=OD 570650 nm−OD  nm treated cellsOD 570650 nm−OD  nm untreated cells×100.

For crude extract and nanoformulations, the determination of MCC was defined as the lowest concentration that inhibited less than 20% of cell viability.

### 2.11. Data Visualization and Statistical Analysis

Data visualization and statistical analyses were performed in RStudio (Version 4.3.2; Posit Software, PBC, Boston, MA; URL https://www.posit.co/). Box and bar plots were created using *ggplot2* and *tidyverse* packages (URL: https://ggplot2.tidyverse.org). The *tidyverse* suite for data management and *multcomp* package for multiple‐comparison tests were also used. Three technical replicates per concentration were used per comparison to 100% MPS for the positive control and untreated cells for the negative control. A one‐way ANOVA model was fit to the data. Treatment‐vs.‐control contrasts were evaluated using the single‐step Dunnett procedure, which controls the family‐wise error rate for all *k* comparisons against a single reference. Adjusted *p* values < 0.05 were deemed statistically significant. Data are visualized as box‐and‐whisker and box plots (*n* = 3 per group). For crude extract and nanoformulations, the determination of MCC was defined as the lowest concentration that inhibited less than 20% of cell viability.

## 3. Results

### 3.1. Preliminary Screening of Anti‐*Acanthamoeba* Activity of Extracts Against Trophozoites and Cysts

Preliminary screening of antiparasitic activity of the plant extract against the trophozoites and cysts was conducted at concentrations of 4 mg/mL and 8 mg/mL, respectively (with the final DMSO concentration of 2% for trophozoites and 4% for cysts). Table 1 shows the percentage of cell inhibition (mean ± SD) of trophozoites treated with the extracts ranging from 7.70 ± 4.18% to 95.11 ± 1.05%, while inhibition against cysts ranged from 19.06 ± 4.56% to 74.76 ± 2.64%. Extracts were selected for further study if they demonstrated ≥ 90% growth inhibition (mean ± SD) of both trophozoites and cysts when compared to CHX, the positive control. Results showed that *A. triangularis* WU19001 trophozoites were inhibited more than 90% (95.11 ± 1.05%, 89.76 ± 0.31, and 89.73 ± 1.02) when treated with crude *A. muricata* at 4 mg/mL (Table 1). It was noted that crude *A. muricata* possesses the highest trophozoite inhibition against *A. triangularis* WU19001 (95.11 ± 1.05%), followed by *A. castellanii* ATCC50739 (92.32 ± 1.55%) and *A. polyphaga* ATCC30461 (89.74 ± 1.83) as compared to the other *Acanthamoeba* spp., which exhibited less than 90% cell inhibition (Table 1). On the other hand, none of the *Acanthamoeba* cysts showed inhibition more than or equal to 90% growth inhibition at 8 mg/mL (Table 1)*.* Hence, because *A. muricata* showed ≥ 90% trophozoite growth inhibition, it was chosen to further determine the MIC values against all *Acanthamoeba* spp. The higher concentrations were tested from 256 mg/mL to least 0.025 mg/mL with *A. muricata* crude extract.

**TABLE 1:** Percentage of *Acanthamoeba* inhibition of *A. muricata* crude extract against chlorhexidine on trophozoite and cyst forms.

Treatment	Inhibition (%, mean ± SD)			
	Trophozoites (cysts)			
	*A. castellanii* ATCC30010	*A. castellanii* ATCC50739	*A. polyphaga* ATCC30461	*A. triangularis* WU19001
*A*. *muricata*	45.91 ± 6.62 (15.60 ± 3.92)	92.32 ± 1.55 (61.01 ± 3.30)	89.74 ± 1.83 (19.06 ± 4.56)	95.11 ± 1.05 (55.15 ± 0.76)
Chlorhexidine	100.00 ± 0.00 (57.73 ± 9.39)	100.00 ± 0.00 (100.00 ± 0.00)	100.00 ± 0.00 (70.00 ± 2.82)	100.00 ± 0.00 (97.73 ± 1.12)

*Note:* Extracts: trophozoites at 4 mg/mL; cysts at 8 mg/mL; CHX: trophozoites at 0.008 mg/mL; cysts at 0.032–0.128 mg/mL; WU, Walailak University Culture Collection.

Abbreviation: ATCC, American Type Culture Collection.

Furthermore, Table 2 shows that the anti‐*Acanthamoeba* activity of ethanolic extracts of *A. muricata* compared to CHX possessed a stronger potential effect on trophozoites (MIC 4 to > 4 mg/mL) than cyst (MIC > 8 mg/mL). It was noted that *A. muricata* leaf extracts exhibited strong antitrophozoite activity against *A. triangularis* WU19001 at MIC 4 mg/mL, and AME also exhibited positively *A. polyphaga* ATCC30461 and *A. castellanii* ATCC50739 trophozoites at MIC 4 mg/mL. The percent inhibition of *A. muricata* plant extracts represented the promising effect on *Acanthamoeba* species; the compounds from this plant would be further studied.

**TABLE 2:** The MIC and MPC values of quercetin nanoformulation against *Acanthamoeba* sp. trophozoites and cysts.

Treatment	MIC (MPC), mg/mL			
	Trophozoite/cysts			
	*A. castellanii* ATCC30010	*A. castellanii* ATCC50739	*A. polyphaga* ATCC30461	*A. triangularis* WU19001
*A. muricata* extract (AME)	> 4/> 8 (8/16)	4/> 8 (4/16)	4/> 8 (4/16)	4/> 8 (4/16)
Pure quercetin (PQT)	2/8 (2/8)	2/> 8 (2/16)	4/4 (4/4)	0.5/8 (0.5/8)
Nano‐quercetin (NQT)	> 4/> 8 (8/16)	> 4/> 8 (8/16)	2/> 8 (2/16)	> 4/8 (8/8)
Chlorhexidine (CHX)	0.008/> 0.0128 (0.008/> 0.128)	0.016/0.032 (0.016/0.032)	0.016/> 0.0128 (0.016/> 0.128)	0.008/0.064 (0.008/0.64)

*Note:* MIC, minimal inhibitory concentration defined as the lowest concentration with > 90% inhibition; MPC, minimal parasiticidal concentration defined as the lowest concentration with > 99.99% inhibition; WU, Walailak University Culture Collection.

Abbreviation: ATCC, American Type Culture Collection.

### 3.2. GC–MS Profiling and HPLC/NMR‐Based Validation of Quercetin in *A. muricata*

GC–MS profiling of the ethanolic *A. muricata* leaf extract revealed a chemical profile dominated by fatty acid ethyl esters, terpenoids, sterols, and tocopherols, together with several low‐molecular‐weight phenolic compounds, including catechol, methoxyphenols, tyrosol, coniferyl alcohol, and sinapyl alcohol (Supporting Figure 1). Although quercetin was not detectable under the GC–MS conditions employed, this result is expected given its nonvolatile and thermolabile nature, which limits direct GC detection without derivatization. Importantly, the detection of these phenylpropanoid‐derived phenolics strongly supports the biosynthetic presence of upstream precursors within the flavonoid pathway, from which quercetin is biosynthetically derived. These findings therefore justified the use of orthogonal chromatographic and spectroscopic techniques for definitive quercetin identification.

Following GC–MS screening, the crude ethanolic extract was subjected to silica gel column chromatography to selectively enrich semipolar polyphenolic constituents, including flavonoids. Silica gel was selected as the stationary phase due to its strong hydrogen‐bonding interactions with hydroxyl‐rich compounds such as quercetin. Stepwise elution using solvent systems of increasing polarity enabled effective separation of nonpolar lipids and terpenoids from phenolic‐rich fractions. This prepurification step was critical to reduce matrix interference, concentrate the target flavonoid fraction, and improve the resolution and sensitivity of downstream HPLC (1260 Infinity II Agilent Technologies, USA) and NMR (Ascend 600 Bruker BioSpin, Massachusetts, USA) analyses.

HPLC analysis of the flavonoid‐enriched silica gel fraction revealed a well‐resolved peak with RT corresponding to that of an authentic quercetin standard, confirming the chromatographic presence of quercetin in the extract (Supporting Figure 2). The UV–visible spectral characteristics of this peak were also consistent with the diagnostic absorbance pattern of quercetin, characterized by two major absorption bands corresponding to the benzoyl (Band II) and cinnamoyl (Band I) systems of flavonols. These chromatographic findings provided target‐specific chemical evidence for quercetin, warranting structural confirmation using NMR spectroscopy.

The putative quercetin fraction obtained from silica gel and verified by HPLC was subsequently subjected to ^1^H and ^13^C NMR spectroscopy for definitive structural validation. The ^1^H NMR spectrum (Supporting Figure 3) of the isolated compound exhibited characteristic aromatic proton resonances at *δ* 7.73–7.62 ppm, assigned to H‐2′ and H‐6′ of Ring B and *δ* 6.89–6.67 ppm corresponding to H‐5′. Signals at *δ* 6.38 ppm (H‐8) and *δ* 6.18 ppm (H‐6) were consistent with proton environments on Ring A. These chemical shifts closely matched reported quercetin reference values, confirming proton‐level structural correspondence.

The ^13^C NMR spectrum (Supporting Figure 4) further substantiated quercetin identity, showing a diagnostic carbonyl resonance at *δ* 175.94 ppm (C‐4, ring C), along with multiple oxygenated aromatic carbons between *δ* 164.18–161.12 ppm corresponding to C‐5, C‐7, C‐3′, and C‐4′. Additional resonances for quaternary and protonated aromatic carbons across Rings A, B, and C were all in excellent agreement with published quercetin standards. Collectively, both proton and carbon NMR spectra exhibited high concordance with reference quercetin values, providing definitive molecular‐level confirmation of the compound isolated from AME.

### 3.3. Characterization of NPs

**FIGURE 2:**
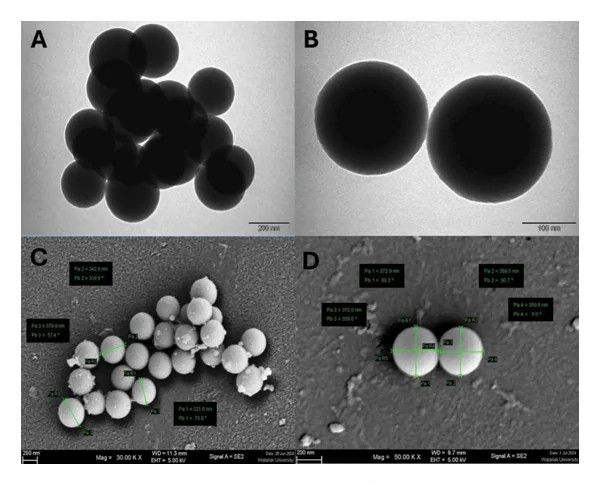
TEM images of quercetin‐loaded SiO_2_ nanoparticles (A, B) and SEM images of quercetin‐loaded SiO_2_ nanoparticles (C, D).

**FIGURE 3:**
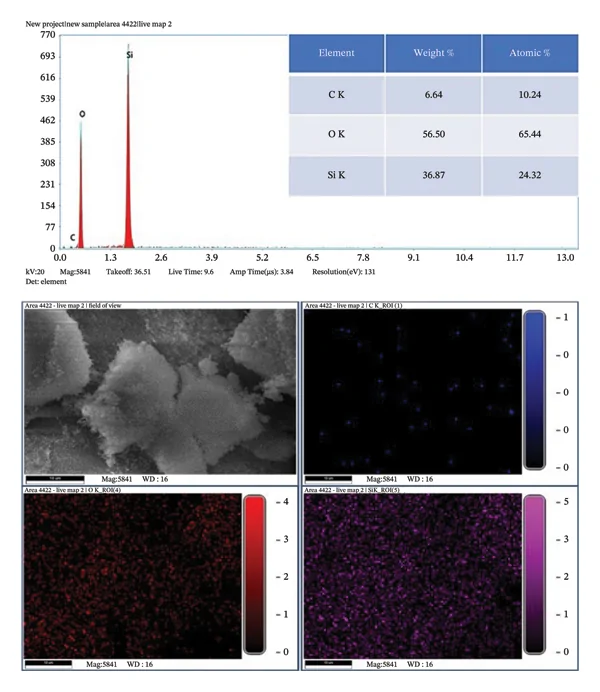
EDX analysis and elemental mapping of quercetin‐loaded silica nanoparticles. C K = carbon K‐shell emission; O K = oxygen K‐shell emission; Si K = silicon K‐shell emission.

**FIGURE 4:**
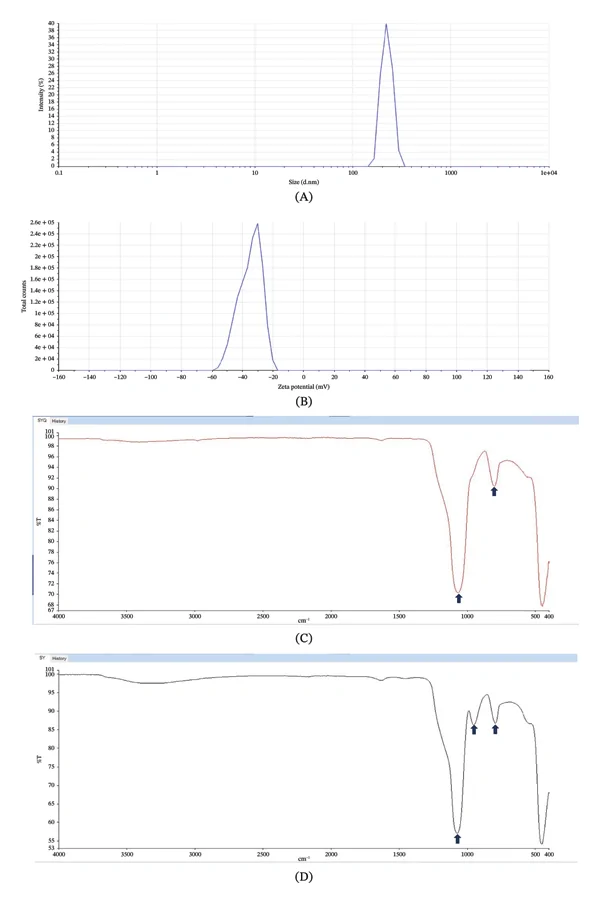
Zeta size (A) and zeta potential (B) of quercetin‐loaded silica nanoparticles, and (C, D) FTIR analysis of nano‐quercetin showing 2 peaks demonstrating the loaded quercetin compound (arrows) or NQT on the nanoparticle blank silica (NSB) nanoparticle correspondingly with 3 peaks.

The SNPs with quercetin were successfully synthesized with a yield of 111.6 mg of encapsulated quercetin nanoformulation using 0.02 g of quercetin compound and 0.2 g of SiO_2_ NPs. The size and morphological examination of the quercetin‐loaded SiO_2_ NPs were performed by TEM and SEM analysis (Figure 2). The smooth surface of synthesized SiO_2_ NPs ensured the complete coating. The morphology of quercetin loaded to SiO_2_ NPs was confirmed by the 3D TEM shown in Figure 3. The EE of quercetin to SiO_2_ NPs was determined using spectroscopy, and the absorbance was measured at 570 nm based on its respective weighed yield and its concentration of SiO_2_ NPs–loaded quercetin compound to calculate the percent encapsulated or entrapped drug [61, 69]. The encapsulated quercetin loaded onto SiO_2_ NPs was successfully found at 82.32%; further encapsulation character is supported by FTIR analysis (Figure 4). Both TEM and SEM revealed that SiO_2_ NPs loaded with quercetin were sphere‐shaped and approximately 160 ± 220 nm in size. Figure 3 shows the EDX spectrum and elemental mapping of quercetin‐loaded SiO_2_ NPs. Results indicated the uniform elemental distribution and incorporation of quercetin into SNPs.

Dynamic light scattering analysis (Figure 4) showed that the average particle size increased from 228.2 nm for blank SNPs to 374.8 nm after quercetin loading, indicating successful encapsulation, drug compatibility between quercetin and nanomaterial used, and nanoformulation stability. The Z‐potential of NSB was −0.26 ± 41.8 mV, whereas that of the NQT was −35 ± 7.43 mV, reflecting a relatively high stable colloidal dispersion. The polydispersity index (PDI) of NSB and NQT was observed to be 0.053 and 0.430, respectively, indicating the monodispersity of the NPs [31].

The drug release behavior of the NQT formulation was evaluated by monitoring absorbance changes over 72 h and expressing the data as percent relative drug release (%RDR), normalized to the initial (0 h) and final (72 h) measurements (Table 3). Across all replicates, absorbance values increased progressively from baseline (mean = 0.0122) to 72 h (mean = 0.0176), indicating continuous release of the encapsulated compound into the surrounding medium. At 24 h, the mean absorbance increased to 0.0152, corresponding to a mean relative drug release of 55.81%. This suggests a moderate initial release phase, likely reflecting the diffusion of surface‐associated or loosely bound drug molecules. By 48 h, mean absorbance further increased to 0.0166, reaching 81.40% relative drug release. This substantial increase indicates a sustained release phase, where drug diffusion from the NP matrix becomes the dominant mechanism. The release trend continued up to 72 h, where all replicates reached 100% relative drug release by definition, confirming that the maximal cumulative release was achieved at this time point. Notably, some variability among replicates was observed during the early phase, with %RDR at 24 h ranging from 41.67% to 70.00%. This variation may reflect differences in NP size distribution, drug loading efficiency, or surface characteristics affecting initial release kinetics. However, convergence of values at later time points (48–72 h) suggests consistent overall release behavior across replicates.

**TABLE 3:** Absorbance‐based relative drug release (%RDR) of NQT formulation over 72 hours.

	Initial	24	48	72	% RDR 24	% RDR 48	% RDR 72
Replicate 1	0.0120	0.0150	0.0164	0.0172	57.69	84.62	100
Replicate 2	0.0128	0.0153	0.0160	0.0169	60.98	78.05	100
Replicate 3	0.0120	0.0150	0.0180	0.0192	41.67	83.33	100
Replicate 4	0.0120	0.0155	0.0159	0.0170	70.00	78.00	100
Mean	0.0122	0.0152	0.016575	0.017575	55.81	81.40	100

*Note:* RDR, percent relative drug release.

### 3.4. MIC and MPC Determinations of Quercetin Nanoformulations

The MIC and MPC concentrations per mg/mL were tested in *Acanthamoeba* spp. In brief, the preliminary concentrations for extract from Table 1 (256 mg/mL to 0.025 mg/mL) were found at 4 mg/mL to 8 mg/mL for antitrophozoite and anticyst activities, respectively. Thus, crude extract and nanoformulations were evaluated at 32, 16, 8, 4, 2, 1 and 0.025 mg/mL (Table 2) and showed 4 mg/mL to > 4 mg/mL inhibition for trophozoites and 8 mg/mL to > 8 mg/mL for cysts. The MIC values indicate the true inhibitory threshold and the higher than value of (>mg/mL) indicated in > 4, > 8, > 0.128 mg/mL are the true MIC values lies in between 4 mg/mL to 8 mg/mL, 8 mg/mL to 16 mg/mL and 0.128 mg/mL to 0.256 mg/mL reflecting a conservative interval in the 2‐fold serial dilution concentrations. Likewise, true MIC values of CHX as the positive control demonstrated the best inhibition against trophozoites at 0.008 mg/mL to 0.016 mg/mL, while anticyst activity was observed at 0.032 mg/mL to > 0.128 μm/mL. Furthermore, the MICs (both for trophozoites and cysts inhibitory concentration) and MPCs (included MTIC and MCIC) are presented in Table 2. The MIC determination is the lowest concentration that demonstrated 90% inhibition of viable growth, and the MPC determination is the lowest concentration that inhibited 99.99% of viable growth.

The MICs and MPCs (included MTIC and MCIC) are presented in Table 2. The results of anti‐*Acanthamoeba* activity showed that PQT had strong inhibitory potentials on trophozoites of *A. triangularis*, *A. castellanii* (both subspecies), and *A. polyphaga*, with corresponding MIC/MPC at 0.5/0.5, 2/2, and 4/4 mg/mL. PQT also affected the cysts of *A. polyphaga* and *A. castellanii* ATCC300010/*A. triangularis* at MIC/MPC 4/4 and 8/8 mg/mL (Table 2). It was noted that NQT possessed a significant effect on *A. polyphaga* trophozoites and cysts (MIC/MPC 2/2 and > 8/16 mg/mL) and *A. triangularis* (MIC/MPC > 4/8 and 8/8 mg/mL) (Table 2), an these results are further pursued to observe the effect of the NQT and PQT on *A. polyphaga* and *A. triangularis* species under SEM when compared to natural AME. Thus, according to the above results, the quercetin‐loaded SNPs improved, with better activity on *A. polyphaga* trophozoite (MIC/MPC 2/2 mg/mL), compared to quercetin‐unloaded SNPs (MIC/MPC 4/4 mg/mL), as shown in Table 2. These results suggested that the potential flavonoid compound, quercetin, could be as effective as other anti‐*Acanthamoeba* agents, especially when combined or encapsulated in nano‐delivery systems. Still, their effectiveness, especially against trophozoites, is inferior to that of traditional agents such as CHX.

Overall, the MIC and MPC data demonstrate that the viability of trophozoites and cysts challenged with the crude AM, nanoformulation, and PQT fractions did not surpass the antiprotozoal activity of the CHX control group. However, ethanolic crude AM fractions and PQT may show more anti‐*Acanthamoeba* potential in 3 species than the nanoformulated quercetin‐SiO2, which is moderate in its activity, whereas CHX still works effectively against *Acanthamoeba* trophozoites. Notably, antitrophozoite activity of NQT in *A. polyphaga*, in both in vitro assays for trophozoites and cysts, indicates an improved delivery of the drug compound to target cells, while CHX did not show any significant growth inhibition against the resistant cystic stage. Likewise, the MIC of AME and PQT may have more potential activity against 4 *Acanthamoeba* spp., but the MPC of PQT showed more specific inhibitory activity compared to AME, while the MPC of NQT showed favorable trophocidal activity in AP (ATCC30461) than AT (WU19001) and AC3 (ATCC30010) and AC5 (ATCC50739), while only the NQT against *A. triangularis* cysts may show inhibition at 8 mg/mL compared to 16 mg/mL cysticidal activity in *A. polyphaga* and both *A. castellanii* species were not exhibited in these concentrations or did not show significant growth inhibition against the resistant cystic stages. Both MIC and MPC values demonstrated that the tested concentrations exhibit targeted anti‐*Acanthamoeba* activity and their respective forms in *A. polyphaga* and *A. triangularis*. Further cellular inhibitory effects in these species were demonstrated in SEM micrographs below.

### 3.5. SEM Study

**FIGURE 5:**
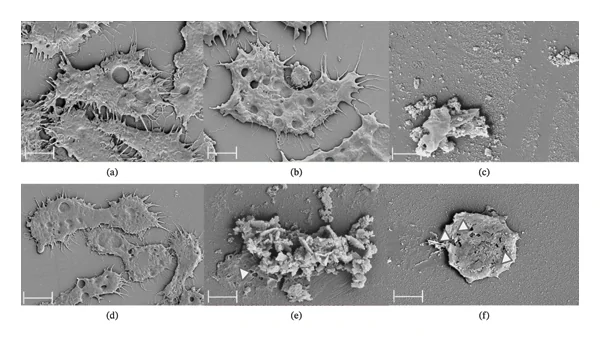
Anti‐*Acanthamoeba* activity of pure quercetin and nano‐quercetin on morphological *A*. *polyphaga* ATCC30461 trophozoites. (a) Untreated cells; (b) negative control (2% DMSO); (c) positive control (0.016 mg/mL chlorhexidine); cells treated with nano‐silica blank (d); nano‐quercetin (e); and pure quercetin (f). White arrowheads indicate perforations in the cell membrane. (Bar scale, 2 μm, Mag. 5000x).

**FIGURE 6:**
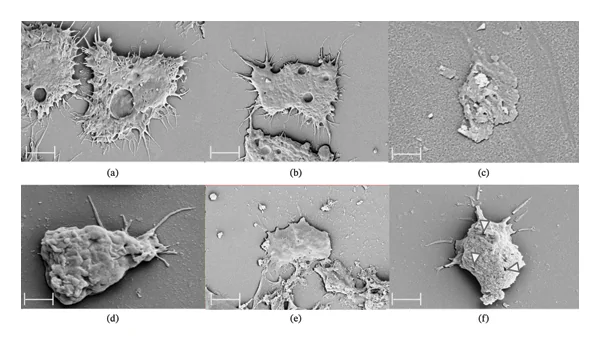
Anti‐*Acanthamoeba* activity of pure quercetin and nano‐quercetin on morphological *A*. *triangularis* WU19001 trophozoites. (a) Untreated cells; (b) negative control (2% DMSO); (c) positive control (0.008 mg/mL chlorhexidine); cells treated with nano‐silica blanks (d); nano‐quercetin (e); and pure quercetin (f). White arrowheads indicate perforations in the cell membrane. (Bar scale, 2 μm; Mag. 5000x).

In both untreated and negative control (2% DMSO), trophozoites of *A. polyphaga and A. triangularis* exhibit the pronounced, crater‐like contractile vacuoles and radiating spiny acanthopodia, an intact cell membrane, and a robust trophozoite size (Figures 5b and 6b). In contrast, CHX 0.016 mg/mL as the positive control in *A. polyphaga* demonstrated loss of cellular integrity as smooth‐surfaced cells with tiny imperforations, indicative of a disrupted membrane without protruding acanthopodia (Figure 5c). Similarly, demonstration of trophozoites cytopathic appearance of PQT (4 mg/mL) manifests a smooth surface with imperforations and shrunken cell sizes, suggesting the mechanism of action of this compound (Figure 5f). This highlights a remarkable finding for quercetin’s strategy in the removal of resistant cyst walls that control its virulence for adhesion. In the untreated, negative control, and NSB, *A. polyphaga* demonstrated numerous acanthopodia radiating from its trophozoites, indicating also its virulence, and maintained vegetative zoites with contractile vacuoles (Figure 5b, d). The NQT‐treated *A. polyphaga* (2 mg/mL) exhibited loss of acanthopodia, removal of the membrane, and deformed cytoplasmic aggregates, indicating an integral cytopathic effect of NQT (Figure 5e). A comparable effect in *A. triangularis* trophozoites was demonstrated with CHX 0.008 mg/mL, shown as a loss of cellular integrity as shrunken, smooth, and flat‐surfaced cells without protruding acanthopodia (Figure 6c). However, the demonstration of the trophozoites’ cytopathic appearance of PQT (0.5 mg/mL) sustained their deformed appearance in a subtle shape with notable severed acanthopodia and an imperforated smooth surface of trophozoites, implying a similar active mechanism of the quercetin compound (Figure 6f). In untreated and negative control (2% DMSO), *A. triangularis* trophozoites demonstrated its salient, numerous radiating acanthopodia from the cell surface and intact contractile vacuoles, indicating also its virulence and maintained vegetative forms (Figure 6b), whereas its trophozoites at 4 mg/mL NSB exhibited an otherwise smooth surface with imperforations and notable morphology; however, few acanthopodia protrusions were observed (Figure 6d). In contrast, the NQT‐treated *A. triangularis* at 4 mg/mL exhibited a similar appearance (Figure 6e) to its positive control, denoting shrunken cell size (Figure 6c) compared to the untreated (Figure 6a) and negative control (Figure 6b), notably, smooth and flattened cellular remnants from dead or damaged trophozoites, a depreciated cellular membrane, and loss of virulent acanthopodia, owing to the cytopathic effect of NQT (2 mg/mL) (Figure 6e). Across *A. polyphaga* and *A. triangularis* trophozoites, treatment with either NQT or CHX was associated with loss of acanthopodia, altered surface morphology, and reduced cellular integrity. These findings support the anti‐*Acanthamoeba* activity of quercetin‐based treatments and warrant further ultrastructural investigation.

**FIGURE 7:**
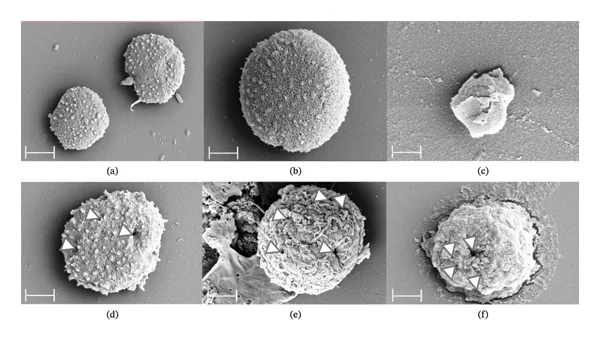
Anti‐*Acanthamoeba* activity of pure quercetin and nano‐quercetin on morphological *A*. *polyphaga* ATCC30461 cysts. (a) Untreated cysts (encystation); (b) negative control (4% DMSO); (c) positive control (chlorhexidine 0.128 mg/mL); cysts treated with nanosilica blank (d), nano‐quercetin (e), and pure quercetin (f) White arrowheads point to “holes,” indicating contact collision onto cyst walls. (Bar scale, 2 μm; Mag: 5000x).

**FIGURE 8:**
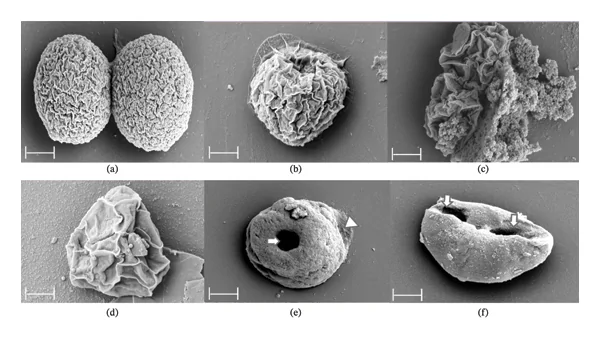
Anti‐*Acanthamoeba* activity of pure quercetin and nano‐quercetin on morphological *A*. *triangularis* WU19001 cysts. (a) Untreated cysts (encystation); (b) negative control (4% DMSO); (c) positive control (chlorhexidine 0.064 mg/mL); cysts treated with nanosilica blank (d), nano‐quercetin (e), and pure quercetin (f). White arrowheads point to “holes,” indicating contact collision onto cyst walls. (Bar scale, 2 μm). White arrows indicate apical ostiole. (Bar scale, 2 μm; Mag: 5000x).

The *Acanthamoeba* spp. cysts show higher concentrations of MIC and the MCIC, and paralleled SEM observations reveal that cysts have also manifested cytopathic effects in *A. polyphaga* ATCC30461 cysts and *A. triangularis* WU19001 cysts, respectively (Figures 7 and 8). In the untreated (Figure 7a), the normal cysts of *A. polyphaga* exhibit a coarse surface with a coarse appearance attributed to its ectocyst with underlying endocyst membranous walls. The normal morphology of *A. polyphaga* cysts was observed in the untreated (Figure 7a), 4% DMSO as the negative control (Figure 7b), and NSBs (Figure 7d). The slight formations of holes in NSBs at a concentration of 8 mg/mL may also indicate a preliminary effect of NP, but the general morphology of the cysts remains unchanged when compared with the untreated and 4% DMSO negative control. Meanwhile, *A. triangularis* cysts exhibit normal wrinkled or creased ectocysts, fibrous ectocysts, and underlying endocysts (Figure 8a). The *A. triangularis* cysts presented a depressed, apical ostiole as the meeting point of cystic walls in 4% DMSO (Figure 8b). In the same negative control, structured adhesion forms the outer margin of the cysts indicated with an arrow. A lower creased appearance was observed in NSBs with consistent triangular morphology (Figure 8d). In the positive control, CHX 0.064 mg/mL was able to inhibit *A. triangularis* with the delayered cystic walls and separation from the parasite’s internal structures, with noticeable shrunken cyst size (Figure 8c). In NQT at 8 mg/mL, the shrunken cyst observed had a smooth surface with an enlarged hole, indicating the total removal of cystic walls and the absence of protruding acanthopodia (Figure 8e). In PQT at 8 mg/mL, a sustained smooth surface was also observed with enlarged double holes and a noticeable deformed shape (Figure 8f), indicating the cyst’s membrane detachment from the cell as a cytopathic effect of the quercetin compound.

### 3.6. Antiadhesion Activity

**FIGURE 9:**
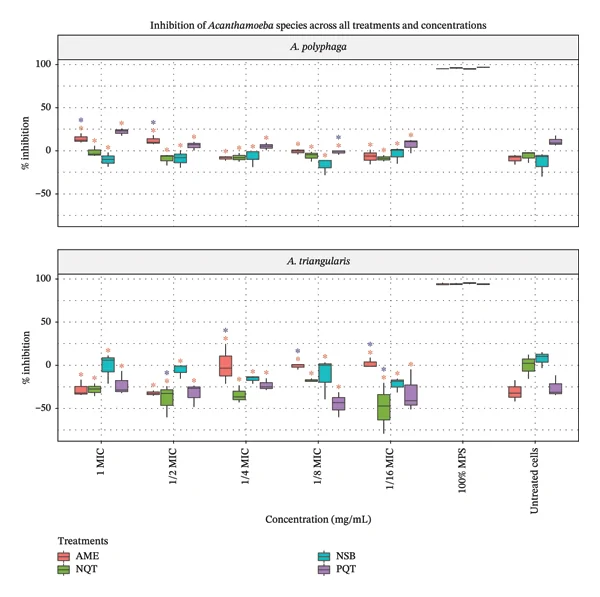
Anti‐adhesion property of *A*. *muricata* crude extract (AME), nano‐silica blank (NSB), nano‐quercetin (NQT), and the unloaded pure quercetin (PQT) against *A. polyphaga* and *A. triangularis*. Treatment concentrations were diluted from 4 mg/mL as 1 MIC, 1/2 MIC, 1/4 MIC, 1/8 MIC, and 1/16 MIC. Data are presented as box‐and‐whisker plots, where the box represents the median and interquartile range (IQR), and whiskers indicate 1.5×IQR. Each condition reflects *n* = 3 technical replicates. Red asterisks (∗) indicate values that are significantly different compared to 100% MPS (positive control). Blue asterisks (∗) indicate values that are significantly different compared to untreated cells (negative control) (*p* < 0.05).

Effects of AMEs on adhesion of *Acanthamoeba* spp. were determined in 96‐well polystyrene plastic plates. The presence of spiny acanthopodia supports the virulence of *Acanthamoeba* parasites and prolongs their viability in the environment. Figure 9 demonstrates that the results obtained at an MIC of 4 mg/mL for *A. polyphaga* and *A. triangularis* were further tested for antiadhesion property (1 MIC, 1/2 MIC, 1/4 MIC, 1/8 MIC, and 1/16 MIC), parallel to SEM findings (Figure 5–8) to observe the effect of NQT. In addition to treatment, observation was done with 100% multi‐purpose cleansing lens solution (MPS) to compare activity with the positive control. The NQT MIC showed less inhibition of cell viability (> 90%) to antiadhesion of cysts in both *Acanthamoeba* spp. tested, wherein trophozoites of *A. triangularis* (Figure 9) demonstrated more activity than *A. polyphaga* (Figure 9) trophozoites based on absorbance at 570 nm, especially for 1 MIC (4 mg/mL) and 1/2 MIC (at 2 mg/mL). The extract together with PQT and NQT substantially reduced the adhesion of *A. polyphaga* trophozoites (Figure 9) to the plastic surface. Approximately a 25% decrease in the trophozoites of *A. triangularis* adhesion to the plastic surface was observed (Figure 9). At the time point, nonencystation of the trophozoites and the destruction of the trophozoites were observed when the cells were challenged with the treatments as shown in Figure 9. The ability to prevent adhesion stands out as a key factor backing up our SEM images and highlighting how this nanoformulation could serve as a promising treatment for infections linked to *Acanthamoeba* spp., particularly when incorporated into solutions for cleaning contact lenses—since the parasite’s stickiness is essentially what makes it so dangerous. When we stacked up the antiadhesion performance of the raw AME against the rest of the nanoformulations, it clearly outperformed NQT. Interestingly, even PQT at its 1 MIC level (4 mg/mL) delivered much stronger antiadhesion activity compared to NQT. These results suggest that fine‐tuning things like how long we let it incubate and the optimal concentration of the NP doses can be effective against *Acanthamoeba* activity and adhesion, ultimately making these nanoformulations even more viable for therapies.

### 3.7. Cytotoxicity of Quercetin and NQT on Vero Cells

**FIGURE 10:**
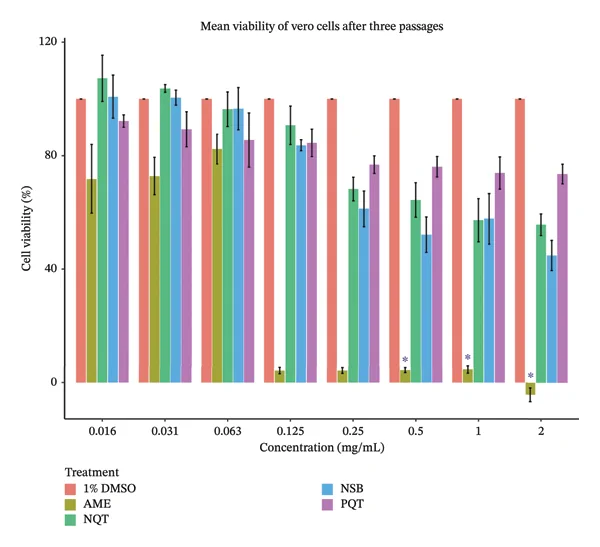
Cytotoxicity tests of unloaded pure quercetin (PQT), nano‐quercetin (NQT), *A*. *muricata* crude extract (AME), and nano‐silica blank (NSB). Data are presented as bar plots with error bars representing SD (*n* = 3 technical replicates). Highest cell viability was demonstrated in NQT.

The cytotoxicity assay was used to determine the cytotoxic effects of quercetin and quercetin encapsulated in SiO_2_ NPs on normal mammalian kidney cells. Vero cells were exposed to varying concentrations of test compounds ranging from 0.016 to 2 mg/mL (Figure 10). No distinct reduction in NQT and PQT was observed. The MCC of *A. muricata* crude extract was 0.063 mg/mL; the others were 0.125 mg/mL. Figure 10 shows that there is high mean viability of normal mammalian kidney cells exposed to nanoformulations (NQT and NSB) and PQT. Individually, a similar trend was observed in each cell passage (8, 9, and 10). These results suggested that the NQT showed consistently less toxicity than *A. muricata* crude extract, even at the highest concentration of 2 mg/mL.

A one‐way ANOVA followed by single‐step Dunnett contrasts was used to evaluate treatment effects at each concentration. Assumption testing showed that variance homogeneity was satisfied for all concentration–passage combinations (Levene *p* > 0.05). Normality of residuals was met in all but one case (Vero‐p10 at 2 mg/mL; Shapiro *p* = 0.015), but ANOVA is robust to minor deviations from normality. Across passages, *A. muricata* crude leaf extract (AME) exhibited significantly lower cell viability than the 1% DMSO control at concentrations ≥ 0.5 mg/mL (Dunnett *p* < 0.05). NQT consistently produced the highest viability values, often exceeding 100% at low concentrations (0.063–0.031 mg/mL), and was significantly less cytotoxic than AME and PQT. NSB showed no cytotoxicity at any tested concentration, indicating that the nano‐silica carrier is biologically inert. PQT produced intermediate viability, higher than AME but generally lower than NQT.

## 4. Discussion

*A. muricata* (guyabano; soursop) is one of the most common tropical trees in the Philippines [34] and Southeast Asia [35]. Several parts of the plant contain constituents with potential medicinal value and phytochemicals [14]. In the present study, the crude extract showed an inhibitory effect against *A. triangularis* trophozoites. However, only quercetin inhibited the cysts. The MIC data also indicated that quercetin was more effective against trophozoites than cysts, as the cyst stage is highly resistant to drugs and other agents [36]. Destruction of the cell membrane was observed when trophozoites were treated with quercetin, while pore formation was noted in cysts, leading to cell membrane disruption. This result aligns with a study in which *α*‐mangostin from *Garcinia mangostana* pericarp inhibited the growth of *A. triangularis* [32]. The study has shown membrane destruction and pore formation in trophozoites and cysts, respectively. This is consistent with the effect of quercetin on *Entamoeba histolytica* by inhibiting energy metabolism and inducing apoptosis through DNA damage, protein kinases, and cell proliferation. This morphological effect could validate the cytotoxic effect of quercetin on *Acanthamoeba* [37].

The percent of drug entrapment is 82.32% using the data mass yield [60]. This percentage of entrapped drug using SiO2 NP may prove the compatibility and stability of the selected SiO_2_ as a nanodrug carrier for quercetin; moreover, the percent of the loaded drug with SiO_2_ NP and as an overall nanoformulation and its dose still exhibited a moderate efficiency on anti‐*Acanthamoeba* activity, indicative of the stable drug delivery that has been carried out to the target cells, especially for trophozoites, as supported by the SEM study. We can also infer from our yield data that the nanoformulation (NQT) may not be a stand‐alone drug against the therapeutic agent but as an adjunct to therapeutic agents that exhibit direct and toxic effect on both target cells and normal cells. Also, based on the data yield that demonstrated NQT with moderate anti‐*Acanthamoeba* activity by quercetin compound alone signifying a limited activity as compared to better activity seen in the crude extract, it may reflect that orchestrated phytocompound interaction is present in AME. Thus, an optimized activity of quercetin can be coactive with other compounds in the plant extract. Nevertheless, we cannot undermine the potency of the NF action as an anti‐*Acanthamoeba* drug showing moderate inhibition. The NQT being an antimicrobial agent for *Acanthamoeba* may have been affected by several pharmacokinetic factors as reflected in its MIC and MPC such as the dose of drug loaded, the loading time, the free drug release, and the stability of the drug compound, which are part of surface functionalization [38] and may implicate its clinical or practical relevance. However, the following factors can still be improved to make the NP stable while ensuring efficient drug delivery and release. The NQT formulation alone still showed a targeted cellular activity and a safer drug formulation in normal cells that are also critical factors in substantiating the pharmacologic value of the NQT intended for inhibition of *Acanthamoeba* spp.

According to Zhou et al. [38], crucial factors in nanoformulation are the colloidal, chemical, and structural stability of SiO_2_NPs, which serve as the primary determinant of their behavior, safety, and efficacy across industrial and biological systems. In their pristine state, bare amorphous possess high surface energy and a dense concentration of reactive surface silanol groups (Si–O–H), rendering them highly susceptible to spontaneous condensation and rapid aggregation [38]. To mitigate these interfacial dynamics, surface functionalization with organosilanes or polymers is required to introduce steric hindrance or modulate the Z‐potential, creating a repulsive energy barrier that maintains a monodisperse suspension [39]. In our results, the negative Z‐potential of NQT, −35 ± 7.43 mV at pH7.4, reflects a negative Z‐potential, exhibiting a strong repulsive electrostatic activity that can impact the prevention of microaggregation of drug particles in the encapsulated NP and provide stability by nonpremature drug release at given time due to its “drug pocketing” property of mesoporous nanosilica. Thus, promoting smooth control of drug release over time dictates the physical stability of the NP that can impede the drug release behavior [40]. Moreover, since NQT displays a strong negative Z‐potential, this indicates the high shifting behavior from negative to positive potential, hence enhancing the weaker bond interactions which hold the drug and, consequently, accelerate drug release in biological environments [41]. When introduced into complex biological fluids like human plasma, these particles undergo a thermodynamic transformation as proteins adsorb onto their surfaces, creating a “protein corona” that often masks targeting ligands and triggers clearance by the mononuclear phagocyte system [41]. In our results, the nanosilica material loaded with the natural product quercetin displays compatibility between the SNP and the loaded quercetin via electrostatic interactions and the negative Z‐potential, which heavily dictates its release. It is also an advantage that using hydrophilic nanomaterials like mesoporous silica nanomaterials can promote acceptability in biological environments like plasma [41] and act as a nanocarrier for quercetin, a hydrophobic polyphenol [42]. Modern biomaterial engineering addresses this via dual‐functionalization architectures such as anchoring zwitterionic groups alongside targeting ligands, which build a protective hydration layer that suppresses nonspecific fouling and preserves cell‐selective targeting in raw plasma [41]. Furthermore, a NP’s stability profile cannot be decoupled from its physical phase and size; transitioning from an amorphous structure to a highly ordered crystalline lattice via high‐temperature calcination significantly increases cytotoxicity, inducing membrane lysis and lipid peroxidation [43]. Similarly, while larger particles remain sequestered within lysosomal pathways, ultrasmall, highly stable particles (< 15 nm) readily penetrate biological barriers and can induce size‐dependent mitochondrial or retinal stress if they accumulate in sensitive subcellular environments [44]. Finally, when utilized as structural additives in polymer nanocomposites, they enhance thermal degradation thresholds and fracture toughness, though this reinforcement collapses at higher weight fractions (10) where local NP agglomeration creates critical mechanical weak points [39].

Although NQT demonstrated improved activity compared with pure quercetin, the magnitude of the enhancement was modest, particularly for trophozoite inhibition, where the improvement was approximately two‐fold. This limited increase may be attributed to quercetin’s intrinsic pharmacodynamic ceiling, with further enhancement depending more on delivery efficiency than on compound potency. Importantly, the MIC values of both quercetin and its nanoformulation remained in the mg/mL range, substantially higher than those of CHX, which exhibits activity in the μg/mL range [8, 10]. While this difference underscores that NQT is not yet competitive with current first‐line therapies, the findings remain therapeutically relevant for two reasons. First, CHX is associated with ocular toxicity, impaired corneal healing, and poor cysticidal performance [8–10], highlighting the need for safer alternatives. Second, the silica nanocarrier improved quercetin solubility, stability, and cellular interaction, demonstrating that biocompatible nanocarriers can enhance natural compounds with otherwise limited aqueous bioavailability [22, 25–27]. Thus, the present results provide a proof‐of‐concept foundation for future optimization of loading efficiency, surface functionalization, and targeted delivery to achieve clinically meaningful potency.

As the SNPs showed an improved targeted drug delivery system, they also demonstrated clear cellular disintegration in the *Acanthamoeba* compared with the free, unconjugated quercetin (PQT) as it did not exhibit highly selective antiamoebic activity against the tested *Acanthamoeba* species [24]. This directly supports the antiamoebic properties of quercetin as it can affect up to 68% of the cells of *A. castellanii* when loaded onto zinc–copper bimetallic NPs or AgNPs [5, 16]. In addition, quercetin forms a macromolecular scaffold that functions as a template for the formation of mesoporous SNPs, which enhances the stability and integrity of the compatibility of quercetin and SNP [45]. This confirms that encapsulating quercetin in a SNP improves its effective concentration and delivery inside the target cell, whether it be a cancer cell or an *Acanthamoeba* trophozoite and cyst, thus amplifying its natural therapeutic mechanism [5].

To improve the nanoformulation using an optimized method for drug loading [69], such as increasing the dose of the drug compound or isolated quercetin, the selection of SiO_2_ as a drug nanocarrier for quercetin may show moderate activity against the protozoan, but the nanoformulation in this study and the working dose were found to be safe as demonstrated by its cytotoxicity activity in Vero cells. This may also suggest an improvement in the level of testing of doses near the MIC values and improve the pharmacokinetics of this nanoformulation. The MIC values of the tested compounds (in the mg/mL range) are substantially higher than those of the standard treatment (CHX), which shows activity in the μg/mL range. With this finding, although CHX exists as a standard treatment, it is also found to be cytotoxic and thereby indicative of a smaller therapeutic index used at μg/mL, and this may still warrant careful use as an active drug due to its harmful effect on normal cells. Considerably, the addition of CHX as a drug can be more efficient and stable if combined with other drugs. Also, the moderate efficiency of NQT against *Acanthamoeba* spp. may be improved or suggested to further develop this nanoformulation for disinfecting agents as it may also show antiadhesion activity as compared with MPS results. Moreover, this nanoformulation can be suggested as a promising treatment as a combined therapy or an adjunct therapy.

In our study, the choice of a less toxic and safe nanomaterial for drug delivery, like SNP, qualifies as a good option because its physicochemical features can be optimized and improved when encapsulated with a biocompatible compound. The improvement in these aspects and Z‐potential make hard NP like SNP as a carrier for quercetin. The silica nanomaterial in small quantity showed lower toxicity in normal cells and was fit to be assessed with its activity against targeted cells in in vitro analysis like *Acanthamoeba* spp. Quercetin, along with most flavonoids, is known to possess antimicrobial activities against bacteria, fungi, and parasites. Its mode of antiparasitic action against the common protist *E. histolytica* involves a combination of factors, such as the inhibition of energetic metabolism, cell proliferation, and protein kinases. Furthermore, several flavonoids have been reported to damage DNA and induce apoptosis in *E*. *histolytica* [37]. This may explain the pore formation in cysts and the destruction of cell membranes observed in the study. Quercetin also exhibits mechanisms, such as antioxidative, anti‐inflammatory, and antiproliferative pharmacological activities, as demonstrated in several in vitro and in vivo studies [54]. This study supports the proteolytic activity of quercetin, which can be enhanced by NPs to facilitate targeted drug action. The demonstration of the effects of a quercetin NP in both trophozoites and cysts of *A. polyphaga* and *A. triangularis* represents the final destination of internalized nanoformulations, leading to overall cellular disintegration as observed in the SEM findings. These results may align with the study of Kolesova et al. [55], which demonstrated albumin NP activity targeting endosomal proteolytic pathways. Similarly, SNPs enhanced surface interactions with *Acanthamoeba* trophozoite membranes and disrupted cell walls, producing a synergistic effect of quercetin and SNP combinations. Generally, quercetin is known to be safe and well tolerated by the body with dosage that is not more than 1000 mg/day. However, significant considerations must be given attention regarding the possible effects of quercetin [46, 47]. Studies have shown that low to regular amount of quercetin can protect the kidneys from the effects of high level of drug dosage and toxins like heavy metals, but high level of dosage in oral administration can show risks of damage to individuals with compromised kidney function and mild gastrointestinal obstructions [48, 49]. In addition, intravenous administration of quercetin shows effects on the cardiovascular system and central nervous system as it can be linked to occurrence of severe headache, tingling sensations, and heart palpitations [46, 47].

Interestingly, the crude extract exhibited stronger antiadhesion activity than the purified quercetin nanoformulation. This discrepancy suggests that the antiadhesive effect of *A. muricata* may not be driven by quercetin alone but rather by synergistic interactions among multiple phytochemicals present in the crude extract [14]. Compounds such as acetogenins, alkaloids, and flavonoid derivatives—previously reported in *A. muricata*—may contribute additively or synergistically to surface‐level interference with *Acanthamoeba* adhesion [14, 15]. Purification and NP loading may also reduce activity due to loss of coactive constituents, suboptimal EE, or slow‐release kinetics that limit the immediate availability of quercetin during the 24‐h adhesion window [22, 29–31]. These findings highlight that, while nanoformulation improves quercetin’s stability and antitrophozoite activity, crude extracts may retain broader antiadhesive functionality due to their composite phytochemical profile. Future work should therefore evaluate multicompound nanoencapsulation or co‐loading strategies to preserve synergistic effects while improving delivery.

The interest of the current study accentuates to test the effectivity of the crude extract against *Acanthamoeba* spp.; hence, we find light in using NPs with contingent knowledge to improve drug delivery, facilitate targeted delivery to cellular, tissue, or even molecular targets in biomedicine, and enable significant interventions in biological and clinical diagnostics and therapeutics. Thus, giving conception in the significance of phytocompounds in nanopharmaceuticals and biomedical diagnostics and treatments in nanosystemic level. Mitchell et al. [50] cited that, generally, NPs as vehicles can potentially enhance the drug or compound stability and provide solubility of encapsulated cargos implying drugs or compounds with limited or poor solubility; the NP design can uphold the transport across biological membranes and prolong circulation times to increase safety and efficacy. For these reasons, NP applications diversify to biokinetics studies, in which the yielded results promise to capacitate its understanding in vitro and in small‐animal models. Thus, we consider that in vitro bioassays like *Acanthamoeba* cellular forms should be nano‐directed target cells with less toxicity to normal host cells. Furthermore, the selection of the NP type is crucial prior to its synthesis. Mitchell et al. [50] briefly described its three classes are the *polymeric* (i.e., polymersome, dendrimer, or nanospheres), the *inorganic* (i.e., gold, iron, and silica) and the *lipid-based* NPs (i.e., liposome, nanoemulsions, and lipid NPs) and their general features and applications, including the pros and cons of cargo, delivery, and patient response. Silica and other inorganic NPs have unique electrical, magnetic, and optical properties. Their flexibility due to its variability in size, structure, geometry, tunable pore size, and theranostic applications are among their advantages; however, they may also have limitations due to their toxicity and solubility. Moreover, SNPs and other inorganic NPs can satisfy requirements that limit organic nanomaterials due to its good biocompatibility, inertness, and stability. The mesoporous SNPs showed affirmative results in genetic and drug delivery [50]. The mean NP size of quercetin‐loaded SNPs is not greater than 374.8 nm; therefore, we can infer that their size in biosystems can enhance the mode of action, especially in transdermal drug delivery and as edge activators [51]. The exterior flexibility, NP hard surface, and mesoporous size provide the inert SNP as attuned nanomaterial for compound encapsulation to facilitate quercetin’s poor solubility and stability [50, 51]. A balance in between targeting and its clearance from biosystems is essential, emphasizing that the size can affect enhanced permeability and retention (EPR) effect and that the biophysical mechanistic particle feature focused on ability to target tumors relative to the blood circulation time of applied NPs [51]. This may suggest that nanocompound‐directed activity can be customized relative to its size, and the half‐life of NPs increases as their size range grows from 10 to 100 nm. Therefore, discretion in optical particle sizes should be considered in NP design and its synthesis with their biological organ size interactions, a corresponding balance between its cellular targeting and biosystem clearance as disregarding its hazardous properties [50, 51]. Also, the Z‐potential is essential in NP stability and guided surface modification in the intended or targeted drug delivery; thus, the Z‐potential of quercetin‐SNP, a high Z‐potential (above ±30 mV) in which the quercetin‐loaded SNPs, has −35 ± 7.43, indicating its stability and preventing particle aggregation, whereas lower values can lead to instability and aggregation that can thereby limit its distribution and drug delivery [51, 52]. The character of Z‐potential can be surface charges deployment and influence also loading and release of drug compounds; thus, nanocarriers like SNPs can evade or prevent adverse immunologic reactions ensuring selective interaction only to specific cells while increasing its overall efficacy in drug targeting. A review article by Xiao et al. [53] showed that inorganic nanomaterial may act as a promising therapeutic combination in treating fungal keratitis by using inorganic NPs and photothermal therapy (PTT). Thus, this insight may prelude support that NPs like quercetin‐SNPs as prospect nanodrug carriers can promote clinical PTT interventions in challenging diseases especially those without consensus treatment like *Acanthamoeba* infections, which can relapse and, if not treated, progressive CNS lesions can worsen to GAE and AK [16, 23, 24].

Despite potent inherent qualities of quercetin, such as immune‐modulatory and antiviral properties, Anwar et al. [16] highlights the limited activity due to its low aqueous solubility and rapid clearance in the body. Quercetin is poorly soluble in an aqueous environment. Thus, this does not help in reaching its target tissues. In addition, the study of Lê et al., [24] practically showed that standard‐free quercetin does not have antiamoebic effect in its free form. This showed that if the concentration is not sufficiently delivered to the target site, the drug’s potential intrinsic activity will not be effective. This is where the study of Abdel‐Hakeem et al. [23] showed that the NP can bridge the gap of the drug delivery to the targeted cite for substances like quercetin with poor solubility. The AgNPs facilitated the delivery of an effective amount of quercetin concentration that the free drug cannot do. The NP can encapsulate the drug and facilitate the stable dispersion of quercetin in an aqueous environment. This ensures that the quercetin will not precipitate out while being delivered to the target area with delayed clearance by the body. In an in vivo setup, the study showed effects as an antiprotozoal to *Acanthamoeba polyphaga.* Furthermore, there are also recorded effects of the NP drug delivery on neuroinflammation and endothelial permeability observed in the same setup.

The studies have shown that NPs can be utilized to advance medical applications of phytochemical compounds through drug delivery. However, this validates that quercetin’s potent cytotoxicity is heavily reliant on the action of NPs. The limitation of free quercetin is emphasized due to its low solubility and poor bioavailability which remain challenging to drug delivery as shown by the prior studies [16, 23, 24].

In this study, quercetin was successfully loaded into SiO_2_ NPs, with an MIC of 2 mg/mL for trophozoites and > 8 mg/mL for cysts. Treated trophozoites exhibited irregular cell shapes due to the destruction of acanthopodia. Similarly, *A. triangularis* trophozoites treated with contact MPS displayed a shrunken cell size and cyst‐like shape morphology. Acanthopodia, the primary adhesion structure of trophozoites, are used for surface attachments such as contact lenses [56]. Therefore, the loss of acanthopodia following treatment with crude extracts, quercetin, and MPS could inhibit *Acanthamoeba* adhesion or reduce the amoebic motility on contact lens surfaces. Previous reports have documented potential cytotoxic or pro‐oxidant effects of quercetin in other biological systems, other than Vero cells, which were used in this study. These effects were observed particularly at high concentrations or in nonencapsulated forms. Such variations highlight that quercetin’s safety profile may be context‐dependent, and nanoencapsulation may play a key role in reducing its inherent cytotoxicity [57, 58].

Regarding the SEM study, their encystment is a mechanism for their survival and viability in the environment, especially for nonpathogenic *A. triangularis*, but they can pose a hazard or risk if they contaminate MPSs. Moreover, the profound effect demonstrating smoothness and membranous imperforations may indicate that QT, especially in nanoformulation, can disrupt the mechanisms involved in membrane protein synthesis. The severing and loss of acanthopodia further suggests that the compound can profoundly affect filament and cytoskeletal proteins that contribute to the pathogenicity of the parasite; thus, without acanthopodia, even if present in small numbers, they can attenuate its virulence in disease manifestation such as AK and GAE [59]. Membrane disintegration is demonstrated by observed imperforations and is a sign of compound penetration to weaken the trophozoites’ viability and cellular integrity, emphasizing the cytopathic effects of quercetin and quercetin NPs. According to Siddiqui and Khan [5], actin microfilaments are highly distributed beneath the *Acanthamoeba* plasma membrane, maintaining functional morphology during cell division and forming vegetative cytoplasmic projections that enable tension resistance on adhering surfaces. The loss of acanthopodia, therefore, reflects inhibitory activity, reducing parasite virulence [59]. This suggests potential inhibition of cytoskeletal proteins associated with acanthopodia in trophozoites of *Acanthamoeba* spp., providing a foundation for further development of quercetin NPs derived from *A. muricata* as a potential therapeutic agent against *Acanthamoeba* infection.

In a recent study, quercetin‐conjugated silver nanoparticles (QAgNPs) exhibited potent in vitro amoebicidal activity against *A. castellanii* ATCC 50492 and showed minimal cytotoxicity in vitro against the human keratinocyte HaCaT cell line [16]. Unlike quercetin alone, QAgNPs effectively inhibited both encystation and excystation of *A. castellanii* after 72 h at 30°C, suggesting that QAgNPs possess superior anti‐*Acanthamoeba* activity compared to quercetin. The killing effect of these compounds and extracts on *Acanthamoeba* spp. is promising, as only low doses are required to target both trophozoite and cyst stages. Additionally, these compounds and plants are widely distributed across various environments, making them easily accessible. The ability of QT‐AgNPs to prevent the morphological transformation of trophozoites into cysts, and vice versa, is of immense importance for drug development against *A. castellanii*. Moreover, the amoebicidal activity of encapsulated quercetin in SNPs exhibits an improved targeted drug delivery with reduced cytotoxic effects in Vero cells. Quercetin has been known to have a cytotoxic effect if used alone or as a pure compound [35]; thus, this study designed a compound (quercetin) encapsulated in SNPs to improve its drug delivery and reduce its toxicity, similar to studies with periglaucine‐A and betulinic acid encapsulation of poly(DL‐lactide‐co‐glycolide) NP [60].

The present study demonstrated that PQT and silica‐nanoformulated quercetin exhibited measurable trophozoiticidal and, to a lesser extent, cysticidal activity against multiple *Acanthamoeba* species, with nanoformulation improving activity against selected strains such as *A. polyphaga*. However, their potency remained substantially lower than that of CHX, which achieved inhibitory effects at concentrations several orders of magnitude lower. This observation is consistent with the broader literature, where CHX and polyhexamethylene biguanide (PHMB) remain the most clinically supported agents for AK [61], while newer compounds and repurposed drugs generally possess stronger preclinical than clinical evidence. Similar to other emerging candidates such as miltefosine, voriconazole, and isavuconazole [70], quercetin demonstrates in vitro activity but lacks the translational evidence required to establish therapeutic superiority or clinical utility. Quercetin is not currently comparable to established anti‐*Acanthamoeba* agents such as PHMB and CHX, which are supported by clinical keratitis data [71, 72]. In its free form, quercetin demonstrates limited efficacy, with studies reporting negligible antiamoebic activity or only partial trophozoite reduction without consistent inhibition of encystation or excystation [24]. However, nanoformulation appears to enhance its bioactivity. Quercetin‐based nanocomposites, including QAgNPs, have shown improved in vitro anti‐*Acanthamoeba* effects compared to the free compound [16]. Although still preliminary, these findings support quercetin as an experimental candidate, where formulation‐driven optimization may improve its therapeutic potential against *Acanthamoeba*, warranting further investigation in NP‐based delivery systems. Nevertheless, the low cytotoxicity profile, demonstrated antiadhesive properties, and enhanced delivery afforded by SNPs suggest that NQT may have value as an adjunctive therapeutic, prophylactic contact lens‐care additive, or lead compound for future anti‐*Acanthamoeba* drug development. Collectively, these findings support the growing body of evidence that natural product–derived nanoformulations represent promising additions to the anti‐*Acanthamoeba* pipeline, although established biguanides continue to represent the benchmark against which emerging agents should be evaluated.

AK is an increasingly prevalent issue among contact lens users [62]. Contact lens contamination by *Acanthamoeba* depends on several factors, such as the lens material, type of lens care solution, and cleaning procedures [5]. The use of disinfectant solutions for contact lenses has been linked to infections by *Acanthamoeba* [63, 64]. MPSs have been found ineffective in eliminating *Acanthamoeba* trophozoites, cysts, bacteria, and fungi. Therefore, incorporating broad‐spectrum antimicrobial agents into MPS is essential [65].

The adhesion of *Acanthamoeba* to the host is a critical step in pathogenesis and infection [66]. *Acanthamoeba* trophozoites exhibit a stronger tendency to adhere to contact lenses than cysts [67] due to their spiny surface structures, known as acanthopodia, which are essential for amoeboid movement. As a result, antiadhesion strategies are considered a promising approach to combat *Acanthamoeba* infections, especially in designing drugs in the nanoscale. Furthermore, therapeutic intervention focusing on antiadhesion activities that potentially control or reduce *Acanthamoeba* virulence may further reduce the disease burden on humans and other animals [68, 69].

In this study, the synthesis of compounds from plants into nanoformulations has been demonstrated using quercetin encapsulation of SNP to enhanec their anti‐*Acanthamoeba* activity. The findings of this study provided the therapeutic significance of the nanoformulation for herbal constituents such as quercetin and other natural products because of the reduced toxicity in normal cells, improved drug distribution, and efficient drug delivery to *Acanthamoeba* spp. cells. In essence, the synthesis of herbal drugs in nanoencapsulation is one of its advantages in pharmacologic value, thereby augmenting the practical or clinical relevance of nanoformulated natural products, especially as promising chemotherapeutic or chemopreventive agents. Specifically, quercetin‐SNPs exhibited a targeted cytopathic effect on the parasite by reducing the pathogenicity and virulence of *A. polyphaga* and *A. triangularis*, without toxicity to normal cells. All these findings are noteworthy from the perspective of natural products, as prospecting plant‐derived compounds such Quercetin from *A*. *muricata* increases its medicinal value. The anti‐*Acanthamoeba* potential of quercetin NPs from natural crude extracts like in *A. muricata* represents an interesting foreseeable lead in drug formulation of nanomedicines with specific targeted drug delivery, highlighting their potential as medicines of the future.

## 5. Limitations of the Study

This study presents important findings on the anti‐*Acanthamoeba* potential of quercetin and its silica nanoformulated derivative. However, it also acknowledges limitations of the study that may be considered by future researchers of anti‐*Acanthamoeba* agents. Firstly, all experiments were conducted exclusively in vitro. It did not deduce the observed antiparasitic effects in an in vivo condition. Also, the study did not assess the biological interactions of *Acanthamoeba* within ocular tissues. It also did not analyze immune responses or the pharmacokinetic behavior of the nanoformulated compound. In addition, the study utilized a limited variety of *Acanthamoeba* strains and genotypes, causing limited known genetic diversity. The substantial variation in virulence, drug susceptibility, and encystment biology among genotypes may establish generalizable efficacy.

On a different aspect, the MIC and MPC values for both PQT and NQT remained relatively high compared with those standard anti‐*Acanthamoeba* agents, such as CHX. This suggests that although nanoformulation enhances certain properties of quercetin, its potency remains inferior to current treatment options and requires further optimization. The study acknowledges the possibility of variability in NP preparation, including differences in loading efficiency, particle stability, and release kinetics, which may influence experimental outcomes.

Given these limitations, future studies must include in vivo validation, expanded testing across additional *Acanthamoeba* genotypes, evaluation in contact lens–related models, and optimization of nanoformulation parameters to reduce MIC values, enhance cysticidal activity, and improve NP stability. Addressing these gaps in future studies will strengthen the translational potential of quercetin‐loaded SNPs as a candidate for anti‐*Acanthamoeba* therapeutics or contact lens disinfecting agents. However, further optimization and validation, including in vivo studies and formulation refinement, are required before therapeutic application can be established.

## 6. Conclusion

This study demonstrated that AME effectively inhibited the growth of *A. triangularis* trophozoites and reduced their adhesion to plastic surfaces. Trophozoites treated with AMEs exhibited a loss of prominent acanthopodia, indicating impaired adhesion and motility. Furthermore, quercetin‐loaded SNPs enhanced activity against *A. polyphaga* trophozoites, achieving lower MIC values compared with unencapsulated quercetin while exhibiting reduced toxicity to normal cells. These findings, paralleled with minimal cytotoxicity, highlight the potential of AMEs and quercetin–SNP formulations as novel treatments for the development of anti‐*Acanthamoeba* therapies. In particular, their application in contact lens care solutions used for cleaning, rinsing, and soaking soft lenses could reduce the risk of amoebic contamination. The quercetin SNPs exhibited activity that supports how natural compounds can be refined in nano‐targeted drug delivery, and the minimal cytotoxicity in normal cells shows promising potential as groundwork for nanopathology screening. Yet, these disclosed findings indicate the overall potential nanoformulation such as quercetin SNPs that showed an advantage against *Acanthamoeba* spp., thus suggesting that further optimization and validation, including in vivo studies and formulation refinement, can essentially be substantiated prior to ascertaining their therapeutic applications.

## Funding

The authors received funding from the Plant Genetic Conservation Project Under the Royal Initiative of Her Royal Highness Princess Maha Chakri Sirindhorn, Walailak University Botanical Garden (RSPG‐WU‐14/2567 and RSPG‐WU‐24/2568) and the International Mobility Fund for Research Publication (WU‐CIA‐01803/2024), Walailak University, Thailand. They also thank Project CICECO‐Aveiro Institute of Materials grant numbers UIDB/50011/2020 and LA/P/0006/2020, University of Aveiro, Portugal.

## Conflicts of Interest

The authors declare no conflicts of interest.

## Supporting Information

Additional supporting information can be found online in the Supporting Information section.

## Supporting information


**Supporting Information** Supporting Figure 1: GC–MS profiling of ethanolic extract from *Annona muricata* leaves—chromatogram showing identified phytochemical constituents based on RTs and mass spectra. Supporting Figure 2: HPLC profiling—chromatographic separation illustrating peak distribution of components. Supporting Figure 3: ^1^H NMR spectrum of extracted quercetin—proton NMR signals confirming the characteristic structural features of quercetin. Supporting Figure 4: ^13^C NMR spectrum of extracted quercetin—carbon NMR signals validating the carbon framework consistent with quercetin.

## Data Availability

The data that support the findings of this study are available from the corresponding authors upon reasonable request.
