# Supplementary material for: Anti‐Acanthamoeba Activities of Silica Nanoformulated Quercetin Compound Derived From Annona muricata L. Leaves
Source: Scientifica (Cairo). 2026 Sep 1;2026:5902580. doi: 10.1155/sci5/5902580 (PMC13532571; doi:10.1155/sci5/5902580)
Supplement: Supplementary file 1 [file SCI5-2026-5902580-s001.docx]

**SUPPLEMENTARY FIGURES**

Supplementary Figure 1. GC-MS profiling of ethanolic extract from *Annona muricata* leaves

Supplementary Figure 2. HPLC profiling

Supplementary Figure 3.  1H NMR spectrum of extracted Quercetin

Supplementary Figure 4. 13C NMR spectrum of extracted Quercetin
